# From the Urinary Catheter to the Prevalence of Three Classes of Integrons, *β*-Lactamase Genes, and Differences in Antimicrobial Susceptibility of *Proteus mirabilis* and Clonal Relatedness with Rep-PCR

**DOI:** 10.1155/2021/9952769

**Published:** 2021-06-10

**Authors:** Arezoo Mirzaei, Bahram Nasr Esfahani, Abbasali Raz, Mustafa Ghanadian, Sharareh Moghim

**Affiliations:** ^1^Department of Bacteriology and Virology, Faculty of Medicine, Isfahan University of Medical Science, Isfahan, Iran; ^2^Malaria and Vector Research Group (MVRG), Biotechnology Research Center (BRC), Pasteur Institute of Iran, Tehran, Iran; ^3^Department of Pharmacognosy, Isfahan Pharmaceutical Sciences Research Center, Isfahan University of Medical Science, Isfahan, Iran

## Abstract

**Introduction:**

*Proteus mirabilis* is a biofilm-forming agent that quickly settles on the urinary catheters and causing catheter-associated urinary tract infections. Thus, the spread of multidrug-resistant *P. mirabilis* isolates, with the ability to form a biofilm that carries integron, extended-spectrum *β*-lactamases (ESBLs), and plasmid-mediated colistin resistance genes (*mcr*), represents a severe threat to managing nosocomial infectious diseases. This study is aimed at surveying the prevalence of ESBL, integrase, and *mcr* genes of *P. mirabilis*, isolated from the catheter, to assess the differences in their antimicrobial susceptibility and clonal dissemination.

**Method:**

Microtiter plate assay was adopted to measure biofilm formation. The antimicrobial susceptibility was assessed by the disk diffusion method. Antimicrobial resistance genes (*intI1*, *intI2*, *intI3*, *bla*_TEM_, *bla*_CTX-M_, *bla*_SHV_, *mcr1*, and *mcr2*) were detected by PCR. All of the isolates were characterized by repetitive sequence-based PCR.

**Result:**

From 385 collected catheters in patients admitted to the intensive care unit (ICU), 40 *P. mirabilis* were isolated. All of the isolates could form a biofilm. *Proteus* spp. had intrinsic resistance to tetracycline (95%) and nitrofurantoin (92.5%), which explains the high resistance prevalence. The most widely resistant antibiotic was trimethoprim-sulfamethoxazole (75%). Thirty-three (82.5%) isolates were classified as multidrug resistance (MDR). The prevalence of *intI1* and *intI2* genes was 60% and 25%, respectively. In 6 (15%) isolates, both genes were detected. The most frequent ESBL gene detected in all of the isolates was *bla_TEM_*. Also, no detection for *mcr1* and *mcr2* antibiotic resistance genes was reported. Rep-PCR identified 39(GTG)5 types (G1–G39) of 40 isolates that 38 isolates had unique patterns.

**Conclusion:**

In this study, 82.5% of isolates were MDR with high antibiotic resistance to trimethoprim-sulfamethoxazole. The *intI1* and *bla*_TEM_ were the most prevalent genes in the integrase and ESBL gene family. High diversity was seen in the isolates with Rep-PCR. The increasing rate of MDR isolates with a high prevalence of resistance genes could be alarming and demonstrate the need for hygienic procedures to prevent the increased antibiotic resistance rate in the future.

## 1. Introduction

*Proteus mirabilis* is a charming polymorphic swarming persistent colonizer bacterium that strongly correlates with catheter blockage and urinary stone development. Complicated urinary tract infections (UTIs) at an alarming rate are expanding healthcare challenges, and *P. mirabilis* is the pathogen that must be noticed for causing those particular catheter-associated urinary tract infections (CAUTIs). *P. mirabilis* is a usual cause for complicated UTIs, and it becomes intricate in patients undergoing long-term indwelling urinary catheterization who may develop CAUTIs [1]. Such disorders cause difficulties by the inimitable ability of *P. mirabilis* to create crystalline biofilms, eventually leading to encrusted and blocked catheters [2]. Likewise, they could result in urine retention and reflux and, in severe conditions, septicemia and endotoxic shock in addition to trauma to the urethra and bladder mucosa due to removing the catheter [3–5]. CAUTIs present challenges to treatment strategies for different reasons, including the biofilm formation of *P. mirabilis* on catheters and urolith formation in the bladder and urinary tract, which could be created by multidrug-resistant isolates. Multidrug resistance (MDR) may be mediated by resistance agents located on chromosomes or mutations in a resident gene. However, it may also expand by attaining resistance genes through horizontal transfer [6].

These resistance genes, which are widely present on plasmids, transposons, and integrons, lead to the problem of rapid spread and treatment failure. In the past, most *P. mirabilis* isolates were susceptible to common antibiotic classes, but recent studies in different countries have indicated that antibiotic resistance among *P. mirabilis* isolates is increasing. The *β*-lactam resistance patterns of the *P. mirabilis* isolates have reported the production of various classes of extended-spectrum *β*-lactamases (ESBLs) [7].

The prevailing type of integrons discovered in clinical isolates is class 1 integron, which is highly associated with antibiotic resistance; therefore, they have been extensively studied [8]. Class 2 integrons stay in the second significant type of integrons obtained from clinical isolates. Usually, class 2 integrons are inserted in the nonreplicative transposon [9].

The extension of ESBL indicates a severe threat to managing nosocomial infectious diseases, causing problems for remedial choices of antimicrobial applications. The prevalence of integrons and characterized gene cassettes in Gram-negative bacteria integron-associated multidrug resistance has been investigated. However, it is seldom addressed in *P. mirabilis* [10]. Colistin is one of the last-resort drugs to treat infections caused by MDR Gram-negative bacteria. Although this bacterium is intrinsically colistin-resistant, it can carry plasmid-mediated colistin-resistant (*mcr*) genes and is often overlooked and not screened for mcr. However, this bacterium can serve as a reservoir to transmit these genes to colistin-susceptible bacteria [11].

Various molecular methods, such as repetitive extragenic palindromic PCR (Rep-PCR), ribotyping, and pulse-field gel electrophoresis (PFGE), have been used to assess the genotypic diversity within several bacterial species [12].

Rep-based fingerprinting has been proved to be a fast and reliable tool to differentiate between *Enterobacteriaceae* populations. Rep-fingerprinting uses variations in conserved intergenic palindromic DNA sequences for PCR amplification and an isolate's characterization. These DNA elements are stable, noncoding intergenic repetitive sequences scattered across the genome and act as amplification targets to produce various bands [13, 14].

In this study, the prevalence of integrase genes (*intI1*, *intI2*, and *intI3*) and three ESBL genes (*bla*_TEM_, *bla*_CTX-M_, and *bla*_SHV_), as well as *mcr1* and *mcr2*, was investigated, and the comparison of integron-carrying and non-integron-carrying MDR *P. mirabilis* isolated from the catheter was performed to assess the differences in their antimicrobial susceptibility and clonal dissemination. The clonal relationship for finding the origin of infection of the isolates was also evaluated with Rep-PCR.

## 2. Material and Methods

### 2.1. Bacterial Isolation

In this cross-sectional study from June 2019 to July 2020, 385 nonduplicate catheters (5 days-15 days) from intensive care unit (ICU) patients were collected from various hospitals in Isfahan. The inclusion criteria for this study were that the collected catheters were from the patients without a primary urinary tract infection at admitting time; the minimum time of catheterization was five days.

*P. mirabilis* was isolated from the catheters. For this purpose, we followed the procedures of Mandakhalikar et al., with some modifications [15]. Briefly, catheters were cut into 1 cm segments and dipped in phosphate buffer saline (PBS) to discard loosely attached planktonic bacteria. The sample was transferred to 10 ml PBS and vortexed vigorously for 1 min, then probe-based sonication was directed at 10 W (RMS) for 60-90 seconds, and another round of vortex for 1 minute at the highest speed was repeated. The catheter solution was then cultured on blood agar, Eosin Methylene Blue (EMB), MacConkey agar, and catalase, oxidase, IMViC, and urease are a few examples of conventional biochemical tests that were performed, and *P. mirabilis* suspected colonies were recultured to achieve a pure and single colony, and the ureG gene PCR was performed for genetic confirmation.

### 2.2. Antibiotic Susceptibility Testing, ESBLs_,_ and MDR Detection

Antimicrobial resistance of the isolates to ampicillin-sulbactam (10/10 *μ*g), amoxicillin-clavulanic acid (20/10 *μ*g), ampicillin (10 *μ*g), nitrofurantoin (300 *μ*g), meropenem (10 *μ*g), cefotaxime (30 *μ*g), ceftazidime (30 *μ*g), ceftazidime-clavulanic acid (30/10 *μ*g), cefixime (5 *μ*g), aztreonam (30 *μ*g), amikacin (30 *μ*g), norfloxacin (10 *μ*g), ofloxacin (5 *μ*g), ciprofloxacin (5 *μ*g), tetracycline (30 *μ*g), and trimethoprim-sulfamethoxazole (1.25/23.75 *μ*g) was determined by the disk diffusion method on Mueller-Hinton agar overnight at 37°C. The breakpoints for each antimicrobial agent were interpreted according to guidelines provided by the Clinical and Laboratory Standards Institute (CLSI) standards [16]. Furthermore, MDR isolates were defined, according to Magiorakos et al., when the isolates resist at least one agent in ≥3 antimicrobial classes categorized in MDR isolates, the antibiotic classes that were considered for the definition of the MDR were cephalosporins (cefotaxime, ceftazidime, and cefixime), monobactams (aztreonam), carbapenems (meropenem), aminoglycosides (amikacin), quinolones (norfloxacin, ofloxacin, and ciprofloxacin), folate pathway antagonists (trimethoprim-sulfamethoxazole), penicillins (ampicillin), and beta-lactamase inhibitors (ampicillin-sulbactam and amoxicillin-clavulanic acid) [17]. In addition, the ESBL production of the isolates was detected by the double-disc diffusion synergy test [18].

### 2.3. Biofilm Formation Assay

For this purpose, an overnight culture of *P. mirabilis* isolates was diluted (1 : 100) in TSB to reach the 0.5 McFarland concentration, and 5 *μ*l of the diluted culture was inoculated in each well of polystyrene microtiter 96-well plates (Greiner, Germany). After 24 h incubation of the plates at 37°C, the medium was removed, and the wells were washed carefully with double distilled water (DDW) and fixed by adding 200 *μ*l of ethanol (96%) for 15 minutes. Next, the medium was removed, and the plate was left to dry. The biofilms were stained with 0.1% (*w*/*v*) crystal violet for 20 minutes at 37°C. Next, the biofilm was washed three times with DDW. Then, 200 *μ*l of ethanol–acetic acid (90 : 10) was added to each well, and optical density (OD) was measured at 590 nm with an enzyme-linked immunosorbent assay (ELISA) microtiter plate reader. Each assay was done in triplicate, and the mean absorbance ± standard deviation was calculated for all repetitions of the tests. *Escherichia coli* K12 and *Pseudomonas aeruginosa* ATCC 27853 were used as negative (weakly biofilm-forming) and positive (strong biofilm-forming) control strains, respectively [19].

### 2.4. PCR Amplification of *bla*, *mcr*1, and *mcr*2 Genes and Integrase Gene Detection

DNA was extracted by the phenol-chloroform method described by Sambrook and Russell [20]. The purity and quality of the extracted DNA were evaluated by the NanoDropTM spectrophotometer (OD260/OD280 nm ratio ≥ 1.8). PCR amplification was performed for *bla* genes, which coding for the *bla*_TEM_, *bla*_SHV_, *bla*_CTX-M_, and integron family (*intI1*, *intI2*, and *intI3*) using specific primers (Table 1). We used DNA of *E. coli* isolated and sequenced from Iranian Kidney Transplant Patients as control positive for integrase and ESBL genes, and DNA of *mcr* positive genes was taken from Pasteur Institute of Iran [21].

PCR conditions for all these genes were 3 min at 94°C; 30 cycles of 30 seconds at 94°C, 30 seconds at 54°C, and 30 seconds at 72°C; and finally, 5 min at 72°C. The amplicons were revealed by electrophoresis on a 1% agarose gel with 0.5x TBE (Tris–borate–EDTA) running buffer and subsequent exposure to UV light in the presence of a safe stain.

### 2.5. (GTG)5-Rep-PCR Fingerprinting Technique

**Figure 1:**
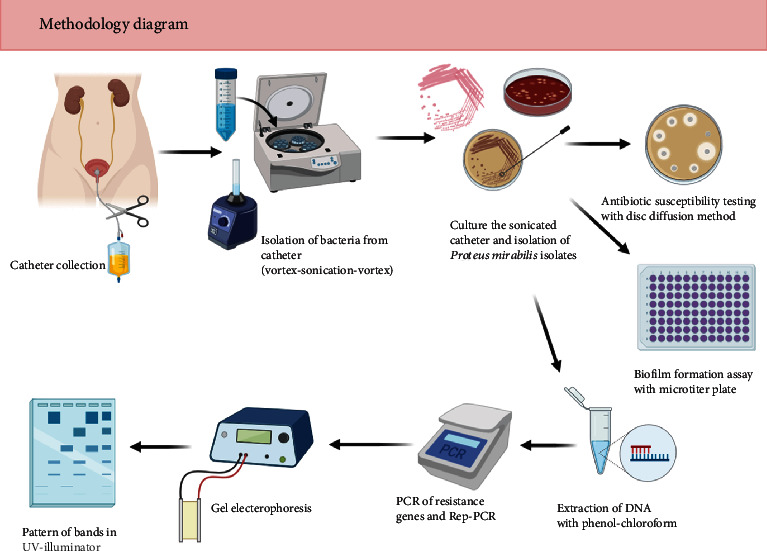
Graphical abstract of methodology.

We followed the methods of (GTG)5-PCR fingerprinting, which was described by Gevers et al. [31]. Analysis of amplicon (GTG)5-PCR patterns and construction of phylogenetic tree were carried out using the curve-based algorithm (Pearson correlation) (Applied Maths, Sint-Martens-Latem, Belgium) to create a similarity scale and an unweighted pair group using arithmetic average algorithm (UPGMA) for cluster analysis, the cut-off value in this GTG_S_ typing predestinated 100% [32]. The band sizes were compared by using a 50 bp ladder. The 15-mer primer (5′-GTGG TGGTGGTGGTG-3′) was used to amplify the repetitive sequences present in the chromosomal DNA of *P. mirabilis*. PCR condition was 3 min at 95°C; 30 cycles of 45 seconds at 94°C, 30 seconds at 50°C, and 45 seconds at 72°C; and finally, 5 min at 72°C. The PCR products were revealed by electrophoresis on a 1.5% agarose gel with 0.5x TBE at 2.5 hours with 65 voltage. This experiment was carried out three times. The graphical abstract of methods which performed in this study is shown in Figure 1.

### 2.6. Discriminatory Index Determination

A discriminatory index (*D*) was calculated to varying levels of similarity index according to the formula. In the formula, *N* represents the number of unrelated strains evaluated, *S* represents the number of distinct types, and *xj* represents the number of strains belonging to the *j*th category, assuming that strains are grouped into mutually exclusive groups.

(1) $$\begin{array}{c} D=1-\frac{1}{N\left(N-1\right)}\overset{s}{\underset{j=1}{\sum }}xj\left(xj-1\right). \end{array}$$

### 2.7. Statistical Analysis

Genotypic diversity among the isolates was calculated using Nei's distances described earlier by Weir [33]. The Rep-fingerprinting methods' discriminatory ability was assessed by calculating Simpson's diversity index, as described previously [34]. Statistical analysis was performed by SPSS Statistics (Version 16). Chi-square or Fisher's exact test was used to determine any statistical association. Statistical significance was regarded as *P* values < 0.05.

## 3. Results and Discussion

From 385 collected catheters in patients admitted to ICU, 40 *P. mirabilis* were isolated. In this study, 72.5% of *P. mirabilis* were isolated from females, and 27.5% were from males. The median age was 42.5 years old (5-75 years). The duration of patients' catheterization, which bacterial isolation was performed, was 5-15 days. Furthermore, the most bacterial isolation was from the ten days of patient catheterization (50%). Patients who had a catheter for 10 to 15 days had moderate to strong biofilm formation; in addition, 50% of patients with short catheterization (7 days) had isolates with weak biofilm development (Table 2).

**Figure 2:**
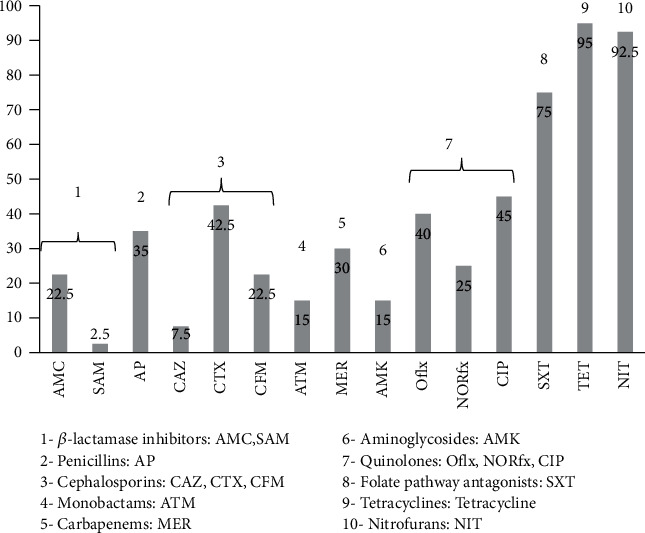
Frequency of antimicrobial resistance of *Proteus mirabilis* strains. Abbreviations: AMC: amoxicillin-clavulanate; SAM: ampicillin-sulbactam; AP: ampicillin; CAZ: ceftazidime; CTX: cefotaxime: CFM: cefixime; ATM: aztreonam; MER: meropenem; AMK: amikacin; Oflx: ofloxacin; NORfx: norfloxacin; CIP: ciprofloxacin; SXT: trimethoprim/sulfamethoxazole; TET: tetracycline; NIT: nitrofurantoin.

The antimicrobial-resistant patterns of isolates are shown in Figure 2. The resistant to tetracycline was 95% followed by nitrofurantoin (92.5%), trimethoprim/sulfamethoxazole (75%), ciprofloxacin (45%), cefotaxime (42.5%), ofloxacin (40%), ampicillin (35%), meropenem (30%), norfloxacin (25%), cefixime and amoxicillin-clavulanate (22.5%), aztreonam and amikacin (15%), ceftazidime (7.5%), and ampicillin-sulbactam (2.5%). *P. mirabilis* is intrinsically resistant to tetracycline and nitrofurantoin which explains its high tolerance to these drugs. Of the 40 *P. mirabilis* isolates, 82.5% were MDR. Furthermore, ESBL was found in 7 (17%) isolates.

All *P. mirabilis* isolated from catheters were able to produce biofilm. Of 40 *P. mirabilis,* twelve isolates (30%) had strong biofilm ability (OD590 nm ≥ 2.5), twenty of them (50%) were moderate biofilm producer (1.5 ≤ OD590 nm < 2.5), and eight (20%) of them had weak biofilm (0.7 ≤ OD590 nm < 1.5) (Table 3).

The frequency of three classes of the integron family is shown in Table 4. The most prevalent gene was *intI1* with 60% (24) of isolates and 25% (10) of isolates had integrase 2 (*intI2*), and no detection for integrase 3; also, 15% (6) of isolates had both integrase 1 and integrase 2. The most prevalent gene in the *bla* ESBL family was observed in *bla*_TEM_ genes with 100% (40) of isolates; 82.5% (33) of isolates had *bla*_CTX-M_ genes and no detection of *bla*_SHV_. Also, PCRs targeting the *mcr*-1 and *mcr*-2 genes revealed no detection for these genes. Also, it demonstrated a significant relationship among resistance to trimethoprim/sulfamethoxazole, norfloxacin, and ofloxacin with a class 2 integron (*P* < 0.05).

**Figure 3:**
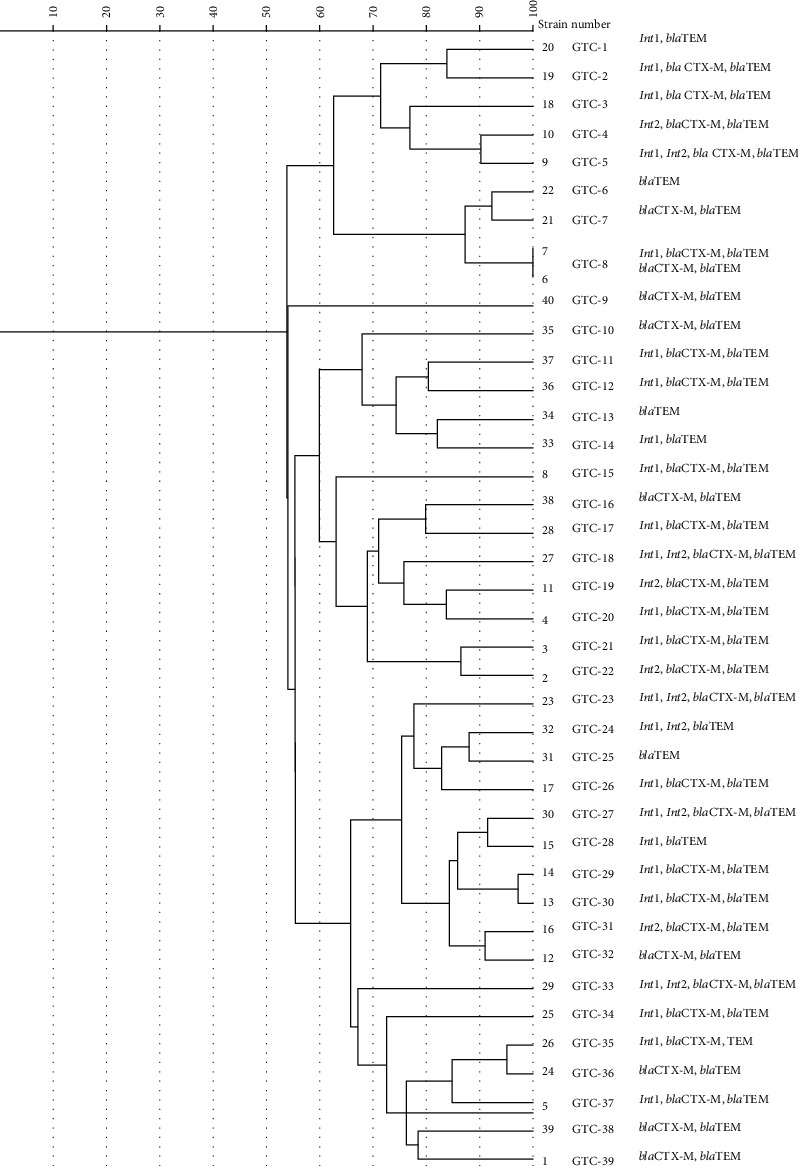
Dendrogram showing genetic relatedness of 40 strains of *Proteus mirabilis* determined by Rep-PCR analysis with Dice similarity coefficient and unweighted pair-group method with average linkages clustering method. Cut-off value: 100%.

Rep-PCR or (GTG)5-PCR fingerprints of 40 isolates generated 7 to 13 bands with the molecular size ranging from 250 bp to more than 1 kb. (GTG)5-PCR amplification identified 39(GTG)5 types (G1–G39) of 40 isolates that 38 isolates had unique patterns (Figure 3). *D* was calculated from a constructed phylogenetic tree, and according to the formula described before, discriminatory power was calculated at 99.8%.

*P. mirabilis* is a common cause of complicated UTI, particularly in patients with functional or anatomical urinary tract abnormalities. Swarming motility can promote the migration of *P. mirabilis* from the periurethral area along the catheter surface into the urinary bladder and initiate CAUTIs in patients undergoing long-term catheterization. There are limited data about the pathogenicity and antibiotic resistance of catheterized *P. mirabilis* in Iran. In this study, we evaluated the molecular characteristics of *P. mirabilis* isolated from CAUTI. The result of the current study showed that all of the isolates had the ability of biofilm formation, being in line with other studies [30, 35]. The severity of biofilm formation depends on the day of catheterization.

The survival and recovery strategy, engrossed by some microbial species in front of rough environmental situations, can form a biofilm, thereby causing antibiotic resistance boosting. The severity of the biofilm-associated antimicrobial resistance of microorganisms is higher than their planktonic form [36]. The present study revealed an increasing growth in antibiotic resistance prevalence in *P. mirabilis* over the past years [30, 37]. The ability of isolates to produce biofilm performs a matrix hindering the penetration of antimicrobial agents through biofilm layers. Moreover, the physiological attributes of microbial cells within biofilms, particularly persister cells, and acquiring of some resistance genes, including integrons family and ESBL genes, could explain biofilm resistance to antimicrobial agents [38]. Although Ojdana et al. [39] found the highest susceptibility to meropenem (100%) in *P. mirabilis* isolates, we found the highest susceptibility to ampicillin-sulbactam (95%); this may be due to differences in the types and amount of antibiotics used in different countries, geographical regions, years of study, and the fact that ampicillin-sulbactam was not used in the antibiotic test, which may explain the difference. The study also revealed a high prevalence of MDR isolates (82.5%), being consistent with the findings of one study by Alabi et al. [37]. In addition, we found a report that contradicts our findings [30] so that we could justify that these differences may be due to differences in the date and location of isolation, as well as differences in the source of isolation (catheter, urine, wound, and blood). In our study, the isolated species were from the biofilm of the catheter causing higher antibiotic resistance, leading to higher MDR percentage.

This study demonstrated a significant association between resistance to trimethoprim/sulfamethoxazole, norfloxacin, and ofloxacin and a class 2 integron (*P* < 0.05). In other words, the presence of the integrase 2 gene in species could lead to resistance to antibiotics, such as trimethoprim/sulfamethoxazole, norfloxacin, and ofloxacin.

Previous studies confirmed that the presence of these elements, mostly class 1 integron, could be considered the evidence for a multidrug-resistant phenotype and associated with the increased frequency of resistance to some antibiotics, such as ciprofloxacin, sulfamethoxazole, and cotrimoxazole [40, 41]. The results indicated a high prevalence of integron-positive isolates and a high antibiotic resistance level regarding the association between integron positive and antibiotic resistance.

In the light of the foregoing, the MDR phenomenon is often associated with the presence of integrons. The coexistence of the ESBL family and integrase genes increases the possibility of the emerging bacteria as a potential carrier of resistance determinants. Integrons can transfer, integrate, express, and distribute resistant agents, facilitating the MDR phenotype of the bacteria. Moreover, these elements entrap several resistance genes belonging to various classes. The prevalence of class 1, 2, and 3 integrons among isolates of *P. mirabilis* investigated in this work is consistent with that reported by others [42–44] and inconsistent with the study conducted by Fursova et al. [45]; this difference could be owing to the time and the source of isolation.

With bacteria's ability to produce ESBL enzymes, the phenomenon of broad-spectrum resistance to *β*-lactam antibiotics is observed and confirmed, but the presence of genes not necessarily leads to the phenotypical appearance of ESBLs as numerous reports have demonstrated [39, 46]. Our analysis revealed such a relationship; therefore, 17.5% of isolates had the phenotypical occurrence of ESBLs. However, the ESBL gene prevalence was higher, which might result in the lack of expression of these genes. According to CLSI reports [47], *Proteus* species bring only the TEM beta-lactamase gene for a long time. At present, the spread of CTX-M is replacing TEM and SHV genes. Hence, the prevalence of *bla*_CTX-M_ is increasing, being in line with our study results [48] with this difference that *bla*_TEM_ rampancy is one hundred percent; this could indicate the spread of ESBL genes among enteric bacteria and acquiring of those genes among the species as well as creation of new and more resistant species that is an alarming issue.

Surveying the epidemiological data over the past years could indicate a dramatic increase in antimicrobial resistance. Overall, the data present continually enhancing resistance to both *β*-lactams and other groups of antibiotics in the *Enterobacteriaceae* family's bacteria [26, 27]. Studies have demonstrated that the genes responsible for the production of CTX-M and TEM *β*-lactamases are more prevalent among tested strains than genes encoding SHV-type *β*-lactamases with no detection report. In this regard, the results of our study are in line with those of previous studies [37, 39, 45].

The steadfast intensify of concurrent resistance to different antibiotic classes significantly reduces the possibility of treatment of infections caused by ESBL producers and MDR isolates [49]. As a result, we aimed to analyze the level of resistance among ESBL-positive and MDR-tested strains. The different percent of resistance in other studies in different parts of the world is most likely due to variations in doctors' antibiotic prescribing prevalence and use in each country, which may be speculative. Other factors that may explain the disparities are the strains and sequence types that are the most prevalent ones in different regions of the world. Increased resistance to antibiotics has been observed in our study in proportion to other studies in Iran and other parts of the world. In this work, 63% of SXT-resistant isolates had the *intI1* gene that was in contrast to further research reporting a 100% relationship between the SXT resistance and the presence of *intI1* [50]. We can explain this issue or the existence of *intI2* or other resistance genes. As indicated by the study conducted by Alabi et al. [37], 100% of MDR isolates had integrase genes, while this work revealed that 72.5% of MDR isolates had integrase genes. Until now, there is no reported existence of the *intI3* gene in *P. mirabilis* isolates, being in line with other studies [37, 43]. As we know, *P. mirabilis* has the intrinsic chromosomal resistance to colistin, but emergence of plasmid-mediated genes for colistin resistance in this bacterium could be a disaster that previous studies reported it to be very low in *mcr1* and *mcr2* genes [11]. In this study, fortunately, there is not a report of those genes. To control the dissemination of these elements and resistance genes, molecular typing of isolates could be sufficient. Molecular typing of *P. mirabilis* is the typical assay conducted to inspect genetic connectedness, discriminate between isolates, and exhibit the source of infection and transmission route with adequate accuracy to recognize the origin of nosocomial outbreaks.

Two efficient methods for *Proteus* characterization at the species level and designation of single strains of *P. mirabilis* are ribotyping and PFGE [51–53]. However, these methods have limitations for use in ordinary clinical laboratories, including being laborious, expensive, and time-consuming. An alternative simple and cost-benefit molecular typing, PCR-based, has been successfully used to identify *P. mirabilis* isolates [54].

The constructed phylogeny tree from Rep-PCR typing effectively identified the genetic relatedness of *P. mirabilis* isolated from the catheter. Rep-PCR (GTG) typing of forty catheter isolates in this study showed 39 types, of which two isolates from one hospital were put in one GTG type (GTG8) with 100% identity, which could be due to the dissemination of the isolates from the hospital environment to patients by personnel or nurses or nosocomial infection, and other isolates were put in single GTG type, which demonstrated high diversity among isolates. Rep-typing results show high discriminatory power, being completely in agreement with the study conducted by Bedenic et al. [55]. The study typing did not confirm the previous research conducted by Michelim et al. [56]. This is most likely due to inconsistencies in the samples and DNA extraction preparation processes, and the most significant reason is the disparity in primers used in this analysis, which differs from our primers.

This GTG8 type, between two isolates, has a similar resistance gene, but the lack of the *intI1* gene has been observed for one of them, which might because the gene release has not yet occurred. The high diversity of isolates in the phylogenic tree was seen in the previous study [30].

The limitation of this study was the lack of a modern technique, such as multilocus sequence typing (MLST) and PFGE, to determine the clonality of isolates due to limited financial sources.

## 4. Conclusions

In this study, 82.5% of isolates were MDR with high antibiotic resistance to trimethoprim-sulfamethoxazole. The *intI1* and *bla*_TEM_ were the most prevalent genes in the integron and ESBL gene family. High diversity was observed in isolates with Rep-PCR. The outcome of persistence MDR strains under antimicrobial selective pressure in the hospital environment will increase both the chance of an advantageous mutation and the opportunity to acquire additional resistance genes. Nevertheless, the existence of the same type of isolates could be an alarming issue for healthcare providers' hygiene conditions to eliminate these infections particularly spread of resistance genes and highlight the importance of sporadically epidemiological studies and methods with high discriminatory power.

## Figures and Tables

**Table 1:** Sequences of primers used in this study.

Gene	Primer sequence (5′-3′)	Product (bp)	Reference
*intI1*	F: GGT CAA GGA TCT GGA TTT CG	483	[22]
	R: ACA TGC GTG TAA ATC ATC GTC		
*intI2*	F: CAC GGA TAT GCG ACA AAA AGG T	789	[23]
	R: GTA GCA AAC GAG TGA CGA AAT G		
*intI3*	F: AGT GGG TGG CGA ATG AG	600	[24]
	R: TGT TCT TGT ATC GCC AGG TG		
*bla*_CTX-M_	F: TTT GCG ATG TGC AGT ACC AGT AA	544	[25]
	R: CGA TAT CGT TGG TGG TGC CAT A		
*bla*_TEM_	F: AGT ATT CAA CAT TTC CGT GTC R: GCT TAA TCA GTG AGG CAC CTA TC	850	[26]
*bla*_SHV_	F: ATG CGT TAT ATT CGC CTG TG	862	[27]
	R: GTT AGC GTT GCC AGT GCT CG		
*mcr1*	F: CGG TCA GTC CGT TTG TTC	309	[28]
	R: CTT GGT CGG TCT GTA GGG		
*mcr2*	F: TGT TGC TTG TGC CGA TTG GA	567	[29]
	R: AGA TGG TAT TGT TGG TTG CTG		
*ureG*	F: AGA ATA TAA TCA ACC ACT GCG TA	514	[30]
	R: CAT TTT GGC TGT ATC CGC TTC		
Rep-typing	GTG GTG GTG GTG GTG	Variable	[31]

**Table 2:** The relationship between the duration of catheterization and the strength of biofilm formation.

Duration of catheterization	Number of isolation (%)	Strong biofilm (%)	Moderate biofilm (%)	Weak biofilm (%)
15 days	4 (10)	3 (75)	1 (25)	0 (0)
10 days	20 (50)	7 (35)	13 (65)	0 (0)
7 days	16 (40)	2 (12.5)	6 (37.5)	8 (50)

**Table 3:** The severity of biofilm and integrase prevalence.

Biofilm	Integrase-1 positive No. (%)	Integrase-2 positive No. (%)	Total no No. (%)	MDR No. (%)
Strong	8 (66.6)	3 (25)	12 (30)	11 (91.6)
Moderate	13 (65)	4 (20)	20 (50)	15 (75)
Weak	4 (50)	3 (37.5)	8 (20)	7 (87.5)
MDR	22 (66.6)	10 (30)	33 (82.5)	—

**Table 4:** Antibiotic resistance pattern of *P. mirabilis* isolates according to integrase 1, 2 positivity.

Antibiotic	No. resistant integrase 1 positive No. (%) *N* = 24	No. resistant integrase 1 negative No. (%) *N* = 16	No. resistant integrase 2 positive No. (%) *N* = 10	No. resistant integrase 2 negative No. (%) *N* = 30	*P* value
AMC	5 (20.8)	4 (25)	3 (30)	7 (23.33)	NS
SAM	0 (0)	2 (12.5)	1 (10)	1 (3)	NS
ATM	8 (25)	3 (18.75)	4 (40)	7 (23.33)	NS
TET	23 (95.8)	15 (93.75)	10 (100)	28 (93.33)	NS
MEM	8 (33.3)	4 (25)	4 (40)	8 (26.66)	NS
NOR	7 (29.1)	5 (31.25)	6 (60)	6 (20)	<0.05
CAZ	9 (12.5)	3 (18.75)	2 (20)	10 (33.33)	NS
Amp	16 (62.5)	6 (37.5)	5 (50)	10 (33.33)	NS
OFX	10 (81)	7 (43.75)	8 (80)	9 (30)	<0.05
CFM	7 (29.1)	2 (12.5)	2 (20)	7 (23.33)	NS
SXT	19 (79.1)	11 (68.75)	10 (100)	20 (66.66)	<0.05
CIP	16 (50)	9 (56.25)	9 (90)	16 (53.33)	NS
NIT	24 (95.8)	15 (93.75)	10 (100)	29 (96.66)	NS
AMK	6 (83)	2 (12.5)	2 (20)	6 (20)	NS
CTX	14 (76)	6 (37.5)	4 (40)	16 (53.33)	NS

NS: not significant.

## Data Availability

The data used to support the findings of this study are included within the article.
